# Validity and Reliability of the Beck Anxiety Inventory (BAI) for Family Caregivers of Children with Cancer

**DOI:** 10.3390/ijerph17217765

**Published:** 2020-10-23

**Authors:** Filiberto Toledano-Toledano, José Moral de la Rubia, Miriam Teresa Domínguez-Guedea, Laura A. Nabors, Blanca E. Barcelata-Eguiarte, Eduardo Rocha-Pérez, David Luna, Ahidée Leyva-López, Leonor Rivera-Rivera

**Affiliations:** 1 Research Unit in Evidence-Based Medicine, Hospital Infantil de México Federico Gómez National Institute of Health, Dr. Márquez 162, Doctores, Cuauhtémoc, México City 06720, Mexico; 2 Facultad de Psicología, Universidad Autónoma de Nuevo León, Dr. Carlos Canseco, 110, Esq. Dr. Aguirre Pequeño, Col. Mitras Centro, Monterrey 64460, Mexico; jose_moral@hotmail.com; 3 Department of Psychology and Communication Sciences, University of Sonora, Blvd. Luis Encinas y Rosales, Col. Centro S/N Hermosillo, Sonora 83000, Mexico; miriamd@sociales.uson.mx; 4 School of Human Services, College of Education, Criminal Justice and Human Services, University of Cincinnati, Cincinnati, OH 45221-0068, USA; naborsla@ucmail.uc.edu; 5 Facultad de Estudios Superiores Zaragoza, Universidad Nacional Autónoma de México, Calz, Ignacio Zaragoza, Col. Ejército de Oriente, Mexico City 09230, Mexico; bareg7@hotmail.com; 6 Servicio Nacional de Sanidad, Inocuidad y Calidad Agroalimentaria (Senasica), Anillo Perif, 5010, Insurgentes Cuicuilco, Coyoacán, Mexico City 04530, Mexico; eduardo.rocha@senasica.gob.mx; 7 Comisión Nacional de Arbitraje Médico, Mitla No. 250-8° Piso, esq. Eje 5 Sur (Eugenia), Vertiz Narvarte, Benito Juárez, Mexico City 03020, Mexico; xeurop@hotmail.com; 8 Centro de Investigación en Salud Poblacional, Instituto Nacional de Salud Pública, Av. Universidad No. 655 Col. Santa María Ahuacatitlán, Cuernavaca, Morelos 62100, Mexico; leyvalop@insp.mx (A.L.-L.); lrivera@insp.mx (L.R.-R.)

**Keywords:** anxiety, BAI, inventory, family caregivers, children with cancer, psychometric evaluation, reliability, validity

## Abstract

Currently, information about the psychometric properties of the Beck Anxiety Inventory (BAI) in family caregivers of children with cancer is not available; thus, there is no empirical evidence of its validity and reliability to support its use in this population in Mexico or in other countries. This study examined the psychometric properties of the BAI in family caregivers of children with cancer and pursued four objectives: to determine the factor structure of the BAI, estimate its internal consistency reliability, describe the distribution of BAI scores and the level of anxiety in the sample and test its concurrent validity in relation to depression and resilience. This cross-sectional study was carried out with convenience sampling. A sociodemographic questionnaire, the BAI, the Beck Depression Inventory and the Measurement Scale of Resilience were administered to an incidental sample of 445 family caregivers of children with cancer hospitalized at the National Institute of Health in Mexico City. Confirmatory factor analysis using the maximum likelihood method was performed to determine the factor structure and exploratory factor analysis using axis factorization with oblique rotation was conducted. The two-, three- and four-factor models originally proposed for the BAI did not hold. The exploratory factor analysis showed a model of two correlated factors (physiological and emotional symptoms). Confirmatory factor analysis revealed a lack of discriminant validity between these two factors and supported a single-factor model. The internal consistency of the scale reduced to 11 items (BAI-11) was good (alpha = 0.89). The distribution of BAI-11 scores was skewed to the left. High levels of symptoms of anxiety were present in 49.4% of caregivers. The scale was positively correlated with depression and negatively correlated with resilience. These findings suggest that a reduced single-factor version of the BAI is valid for Mexican family caregivers of children with cancer.

## 1. Introduction

### 1.1. Impact of Caring for a Child with Cancer on the Family Caregiver

Childhood cancer is a chronic disease that requires continuous medical treatment and frequent hospital readmission and usually affects the quality of life of children and their parents [1,2]. In this situation, parents abandon most of their social and recreational activities and neglect their own health due to the demands of their role as caregivers of the sick child, the patient’s siblings and even other family members [3,4]. Thus, the demands of chronic illness and the effects of providing care influence the quality of life of the family caregiver on the personal, social and vocational levels in addition to increasing their vulnerability to associated emotional and physical disorders, such as depression [5].

A review of published empirical research shows that psychosocial factors are associated with anxiety in family caregivers of chronically ill children [6] and that these caregivers have a higher prevalence of anxiety and distress than the general population [7]. Empirical findings suggest that family caregivers may experience high levels of anxiety, depression, parental stress and caregiver burden accompanied by low levels of family support, resilience and well-being [8]. These disorders are related to cognitive symptoms (forgetfulness, attention deficit, inability to concentrate), physiological symptoms (neurodermatitis, interrupted sleep, changes in appetite), psychosocial symptoms (agitation, loss of interest, fixations) [9] and other adverse family dynamics and personal responses to crises or conflicts related to the caregiver burden [10].

Although anxiety is a normal reaction for a person in a situation perceived to be threatening or critical, symptoms that exceed healthy limits can lead to a severe disorder that affects the overall functioning and quality of life of the family caregiver [11]. The short- and medium-term consequences of anxiety and the importance of timely detection have led to the creation of different instruments for its measurement and evaluation.

### 1.2. Self-Report Instruments for Assessing Anxiety

Among the self-report instruments for assessing anxiety, four are widely applied in the clinic and frequently cited in research studies; all four of these measurement tools were developed for use in clinical populations in the USA. One such instrument is the Hamilton Anxiety Scale [12], which has been used in combination with depression scales to assess the emotional states of the general population and of family caregivers. Another instrument is the Goldberg Anxiety Scale [13], which assesses psychic and somatic anxiety. Both of these instruments have been validated in a Mexican population of family caregivers of adult patients [13,14] and have been used in family caregivers of children with cancer [15]. Similarly, the State-Trait Anxiety Inventory (STAI), developed by Spielberger and Díaz-Guerrero [16], evaluates anxiety as a personality trait and as an emotional state. This last instrument is frequently used in hospital environments and has been validated in Mexico in a sample of caregivers of children in intensive care [15].

The fourth instrument is the Beck Anxiety Inventory (BAI). The BAI was developed to assess symptoms of anxiety independent of symptoms of depression [17]. This approach resulted in better measurement properties than those of the three previously mentioned instruments. The BAI is composed of 21 items. Beck et al. [18] proposed a two-factor model for these 21 items: somatic symptoms and affective-cognitive symptoms. This model has been primarily supported by validation studies [19]. However, a three-factor model (somatic, subjective and panic symptoms) and a four-factor model (neurophysiological, subjective, panic and autonomic symptoms) have also been proposed [20,21]. The BAI has excellent overall internal consistency [17,18,19,20,21] and a high test-retest correlation (*r* = 0.67) [22]. The BAI also demonstrates good concurrent validity, with correlations between 0.78 and 0.81 with the SCL-90 Anxiety Subscale [23], the Hamilton Anxiety Scale [12] and Spielberger’s STAI [17]. Therefore, the available empirical evidence has shown that the BAI is a reliable and valid instrument for measuring symptoms of anxiety [6,24].

### 1.3. BAI Validation Studies

Due to these psychometric properties, the BAI is one of the most widely used instruments in Spanish-speaking countries. Thus, a broad range of studies have validated its measurement properties in different populations, such as the general adult population [25,26], college students [27,28,29], mental health patients [30] and medical patients [31]. Sanz and Navarro [25] and Magan et al. [27] tested a two-factor model, originally proposed by Beck et al. [18], in the general Spanish population and in Spanish university students and both studies obtained empirical support for this model. Another study in a Spanish population of psychiatric subjects conducted by Sanz et al. [30] reported that the BAI had excellent internal consistency (α = 0.90), criterion validity in relation to other measures of anxiety and discriminant validity when distinguishing between anxiety and depression disorders, with a structure of two correlated factors: somatic and affective-cognitive symptoms of anxiety. In Chile, Antúnez and Vinet [28] reported good internal consistency (α = 0.88) and evidence of concurrent validity.

In Mexico, Acosta and García [32] validated the BAI in older adults. Robles et al. [26] reported good internal consistency (α = 0.83), a high weekly test-retest correlation (*r* = 0.75) and high correlations with states of anxiety (*r* = 0.60) and STAI [29] anxiety traits (*r* = 0.59) in the adult population. Tafoya et al. [29] reported good overall internal consistency (α = 0.86) and concurrent validity using the Hamilton Anxiety Scale (*r* = 0.82) in university students with emotional problems. Moreover, a study by Galindo et al. [31] in cancer patients reported good overall internal consistency (α = 0.82) and a four-factor structure (subjective, neurophysiological, autonomic and vasomotor) that explained 46.4% of the total variance and showed a high correlation (*r* = 0.58) with the anxiety subscale of the Hospital Anxiety and Depression Scale developed by Zigmond and Snaith [33].

In Mexico, the BAI has also been applied to family caregivers of chronically ill patients, demonstrating its usefulness in distinguishing different levels of anxiety symptoms [34]. However, its reliability and structural validity in this population have not yet been studied.

The family caregiver plays a key role in pediatric cancer services during a child’s healing process [5,35]. Since oncological diseases have a considerable emotional impact, there is a need to evaluate this impact on family caregivers [36]. To this end, it is important to have a valid and reliable instrument for assessing anxiety in families with chronic pediatric disease [34]. The results of the reviewed empirical studies confirm that the BAI is a measurement tool with good psychometric properties that enables an accurate assessment of anxiety in different populations and contexts. However, its factor structure (one to more than four factors) and the composition of its factors vary from one population to another, as do its cutoff thresholds and norm-referenced scoring [36]. Therefore, it is necessary to determine the factor structure of the BAI, verify its reliability and describe its distribution in the population of Mexican family caregivers of children with cancer. Furthermore, the validation of the BAI in this population opens the possibility of testing the expected relationships between anxiety and the depression and resilience.

### 1.4. Research Problem and Hypotheses

To validate the BAI in Mexican family caregivers of children hospitalized for cancer and to contribute to the theoretical body of knowledge about this measurement instrument, the aim of this study was to analyze the psychometric properties of the BAI. Four objectives were formulated: (1) to determine its factor structure, (2) to estimate its internal consistency reliability, (3) to describe the distribution of its scores and levels of anxiety in the sample and (4) to test its concurrent validity in relation to depression and resilience.

A structure of two (somatic and cognitive symptoms) [18], three (somatic, cognitive and panic symptoms) [20] or four factors (neurophysiological, subjective, panic and autonomic symptoms) [21] was expected as well as excellent or good reliability of the scale and excellent to acceptable reliability of its factors [18,19,20,21,22,23,27,28,29,30,31,33]. The distribution of scores on the BAI was expected not to follow a normal distribution but to be skewed and to show that the caregivers display high levels of anxiety [7,8,9,11,35]. Regarding concurrent validity, the hypothesis was a direct relationship between anxiety and depression with a moderate to high association since both are negative affects [7,11]; in addition, a mixed syndrome of anxiety and depression was expected, in which both types of symptoms overlap [37]. Resilience is a personality variable that protects mental health against the effect of stressors and allows the negative effects of adversity, risks and vulnerability caused by disease to be overcome [38]. Therefore, the expectation was an inverse relationship between anxiety and resilience with a weak to moderate association since personality traits usually have a weak relationship with emotional states [39].

## 2. Materials and Methods

### 2.1. Participants

A nonprobability sampling technique was used. Sampling was performed at the National Institute of Health center in Mexico City through convenience sampling. A total of 445 family caregivers of children hospitalized with cancer voluntarily participated. The criteria for inclusion in this study were as follows: a family caregiver of a child with cancer, over 18 years of age and provision of an informed consent form. All persons who were invited to participate gave their consent. Due to the use of confirmatory factor analysis (CFA), the criteria for determining the sample size were a minimum of 200 participants and 10 participants per parameter to be estimated [40].

Within the primary caregiver population, parents were the subpopulation of interest because they are almost always the only caregivers of chronically ill children [8]. Other types of relatives (e.g., grandparents, uncles or older brothers) appear very exceptionally [13]. Moreover, cancer was chosen because it is the most common chronic disease in children [3,4] and has characteristics of treatment, severity and prognosis that make it a major stressful life event for family caregivers [11].

### 2.2. Instruments

Sociodemographic variables questionnaire (Q-SV) for research in family caregivers of children with chronic diseases [41]. This questionnaire contains 20 questions: 17 questions evaluate social and family variables of the caregiver and three evaluate clinical variables of the pediatric patient.

Beck Anxiety Inventory (BAI). The BAI was developed by Beck et al. [18] and has been validated in the indicated population by Robles et al. [26]. It consists of 21 items that evaluate symptoms of anxiety on a four-point Likert scale ranging from 0 = “not at all” to 3 = “severely.” The anxiety level was scored using ordinal categories: minimal (1–5 points), mild (6–15), moderate (16–30) and severe (31–63) [21].

Beck Depression Inventory, second edition (BDI-II). The BDI-II was developed by Beck et al. [42] and has been validated in the population of Mexican family caregivers of children with chronic diseases [5]. This self-report instrument consists of 21 items to measure symptoms of depression. Participants responded using a four-point rating scale (0 to 3). Higher scores indicate greater depressive symptomology. The BDI-II is composed of two correlated factors: somatic-affective symptoms (12 items: 4, 10, 11, 12, 13, 15, 16, 17, 18, 19, 20 and 21) and cognitive symptoms (nine items: 1, 2, 3, 5, 6, 7, 8, 9 and 14) [12,43]. In the validation study described above, the overall internal consistency reliability was excellent (Cronbach’s alpha = 0.90); likewise, the reliability of the somatic-affective symptom factor was good (alpha = 0.87) and that of the cognitive symptom factor was acceptable (alpha = 0.79) [5].

Measurement Scale of Resilience in Mexicans (RESI-M). The RESI-M was developed by Palomar-Lever and Gómez-Valdez [44] and has been validated in Mexican family caregivers of children with cancer [38]. It consists of 43 items with a four-point Likert-type rating scale from 1 (strongly disagree) to 4 (strongly agree). The RESI-M has a correlated five-factor structure: strength and self-confidence (19 items: 1, 2, 3, 4, 5, 6, 7, 8, 9, 10, 11, 12, 13, 14, 15, 16, 17, 18 and 19), social competence (eight items: 20, 21, 22, 23, 24, 25, 26 and 27), family support (six items: 28, 29, 30, 31, 32 and 33), social support (five items: 34, 35, 36, 37 and 38) and structure (five items: 39, 40, 41, 42 and 43) [45]. Its overall internal consistency was excellent among 330 family caregivers of children with cancer (alpha = 0.93) [38].

The internal consistency reliability values of two validation criteria (total scores and their factors) calculated in the total sample of 455 participants of the present study are shown in the Results section.

### 2.3. Procedure

The primary author of this study administered the measurement instruments to family caregivers. The surveys were conducted in the rooms of the hematology-oncology services of the Hospital Infantil de México Federico Gómez Instituto Nacional de Salud. The interviews lasted approximately an hour. The collection of the sample was carried out for approximately six months, from September 2019 to February 2020.

All the family caregivers interviewed were invited to participate voluntarily; the objectives of the research were explained to them and all of their concerns regarding the study were addressed. The family caregivers who agreed to participate signed informed consent forms and responded to the measurement instruments individually during a single session. Participants did not face any consequences for withdrawing their consent, as specified on the informed consent sheet. Before collecting the answered instruments, the interviewer checked whether there were questions without answers. The participant was asked to respond to any unanswered questions, preventing the occurrence of missing values.

The study complied with the regulations and ethical considerations established for human research currently in effect in Mexico [46] as well as the international guidelines of the Helsinki Declaration [47].

### 2.4. Ethical Considerations

This study is part of “Research Project HIM/2013/019/SSA.1141 Measurement and assessment of resilience in pediatric chronic disease,” which was approved on 16 December 2013, by the Research, Ethics and Biosafety Commissions of the Hospital Infantil de México Federico Gómez National Institute of Health, in Mexico City. While conducting this study, the ethical rules and considerations for research with humans currently enforced in Mexico [45] and those outlined by the American Psychological Association [46] were followed. All family caregivers were informed of the objectives and scope of the research and their rights according to the Helsinki Declaration [47]. The caregivers who agreed to participate in the study signed an informed consent letter. Participation in this study was voluntary and did not involve payment.

### 2.5. Data Analysis

Regarding the first objective, the three models originally proposed for the BAI were tested using CFA since the hypotheses involved the factorial structure of an inventory composed of 21 items (two-, three- and four-factor models). These analyses were computed with AMOS 16 applied to the total sample of 445 participants. The discrepancy function was optimized using the maximum likelihood (ML) estimation method. The errors of point estimations and the significance of each parameter were tested through the bias-corrected percentile method with the simulation of 2000 bootstrap samples [48]. The model fit was evaluated with seven indexes: the chi-square test, normed chi-square (χ^2^/df), goodness-of-fit index (GFI), normed fit index (NFI), comparative fit index (CFI), standardized root mean square residual (SRMR) and root mean square error of approximation (RMSEA).

The following values were considered to indicate a close fit: *p* < 0.05 for the chi-square test; χ^2^/df < 2; GFI, NFI and CFI ≥0.95; and RMSEA and SRMR ≤0.05. The following values were considered to show an acceptable fit: *p* < 0.01 for the chi-square test; χ^2^/df < 3; GFI, NFI and CFI ≥0.90; and RMSEA <0.08 and SRMR <0.10 [49,50]. It was considered that the factorial model presented high parsimony with James-Mulaik-Brett parsimony ratio (PR) values ≥ 0.75. Furthermore, the relationship between fit and parsimony was assessed using parsimonious fit indexes. A parsimonious normed fit index (PNFI) and parsimonious comparative fit index (PCFI) ≥ 0.80 and a parsimonious goodness-of-fit index (PGFI) ≥ 0.70 were considered to indicate a good correlation. A PNFI and PCFI ≥ 0.60 and a PGFI ≥ 0.50 were considered to reflect an acceptable correlation [48]. The goodness of fit between two models was considered to be equivalent when *p* > 0.05 for the chi-square difference test (Δχ^2^); for a normed chi-square difference (Δχ^2^/Δgl) < 2; and for differences in the NFI (ΔNFI), CFI (ΔCFI) and GFI (ΔGFI) < 0.01 [48].

The average variance extracted (AVE) and McDonald’s omega (ω) were calculated. Following the Fornell and Larcker [51] approach, a factor was considered to have convergent validity when its AVE > 0.50 and ω ≥ 0.70. Convergent validity was defined as the degree of confidence that the latent variable could be well measured by its indicators. In turn, two factors were considered to have discriminant validity when their shared variance (square of the correlation between the two factors) was lower than their AVEs. Discriminant validity was defined as the degree of confidence that each factor measured a different content domain. These calculations were performed using Excel 2013.

Due to the poor fit to the data of these three hypothetical models and their important problems in discriminant validity, an exploratory approach was adopted, beginning with an analysis of the properties of the 21 BAI items in the total sample of 445 participants, which was performed using the Rasch methodology in Winsteps software (version 3.7) [52]. From this first exploratory analysis, items with adequate consistency properties were selected for the following factorial analyses: exploration of a new factor structure (in one sample) and test of the new factor model (in another independent sample). Therefore, the selection of items was carried out in the total sample. Once the items were selected, that is, once reliable items were identified, the total sample was randomly divided into two independent subsamples.

The partial credit model (PCM) was used because it handles variables on a polytomous response scale [53]. The estimators analyzed were the indexes of inlier-sensitive fit (infit) and outlier-sensitive fit (outfit), the index of empirical discrimination and point-biserial correlation or correlation between the dichotomized item and the total score of the scale. Infit and outfit values between 0.8 and 1.3 were considered to indicate a reliable and valid item. Values below 0.8 or above 1.3 were considered to indicate an item with poor properties [54]. An item discrimination index value close to one (1) and below 0.9 was considered to indicate discriminative power and a lack thereof, respectively [54]. The point-biserial correlation indicates greater convergent validity as its value increases [54]. Items with tolerance values ≤0.25 were considered to have excessive multicollinearity, which duplicates the estimation error in predictive models and generates cross-loading and correlations between measurement errors; as such, it is advisable to eliminate these variables [54].

Then, the total sample of 445 family caregivers was randomly divided into two subsamples. The factorial structure was explored in one subsample of 224 participants and the factorial model suggested by the exploratory factor analysis (EFA) was tested in the other subsample of 221 participants using CFA. Both subsamples were verified as having equivalent clinical and sociodemographic characteristics using the chi-square test for qualitative variables, the Mann-Whitney U-test for ordinal variables and Student’s *t*-test for quantitative variables. The results verified that this division did not introduce any bias.

Horn’s parallel analysis was performed to define the number of factors [55]. We simulated 2000 samples through data permutation from the correlation matrix. This calculation was performed using the SPSS R 2.4 module. The factors were extracted using principal axis factoring and promax rotation. Configuration matrix loadings below 0.40 were considered to be low [55].

Because the CFA results revealed a lack of discriminant validity between factors, a bifactor model was specified. In this type of model, each item is determined by a general factor, a specific factor and a measurement residual [49]. When evaluating the bifactor model (effects of both specific factors and general factors), either for items corresponding to a specific factor or for the total set of items, the following values were considered to indicate convergent validity: ω ≥ 0.70 and AVE ≥ 0.50 [56]. The evaluation of the contribution of the specific factor (SF) and the general factor (GF) in each content domain was based on six indexes. Significant measurement weights (*p* > 0.05) with moderate or high effect sizes (0.30 ≤ λ_SF or λ_GF ≤ 0.70), average variance extracted (AVE_SF or AVE_GF) values between 0.25 and 0.50, as well as explained common variance (ECV_SF or ECV_GF) and omega hierarchical coefficient (ωh_SF and ωh_GF) values between 0.30 and 0.70 were considered to reflect a substantive contribution. Non-significant weights or small or trivial effect sizes (λ < 0.30), AVE < 0.25 and ECV and ωh < 0.30 were considered to indicate a deficient contribution. On the other hand, significant weights with very large effect sizes (λ > 0.70), AVE > 0.50 and ECV and ωh > 0.70 were considered to indicate an excessive contribution [56]. Finally, the relationship between the number of items and the number of factors was assessed by the percentage of uncontaminated correlations (PUC), which must be less than 0.70 for the bifactor model to be an adequate representation of the interrelationships of the items. If the ωh_GF is greater than 0.70, the PUC must be less than 0.60 [56].

The measurement invariance across the two subsamples was tested for the final model (the one with the best properties). Five models nested in constraints were specified: unconstrained, measurement weights, measurement intercepts, structural covariances and measurement residuals. Parameter equivalence between the two subsamples within each nested model was proven using the Z-test. The goodness of fit of each nested model was valued through six indexes: the chi-square test, χ^2^/df, non-normed fit index (NNFI), CFI, RMSEA and SRMR. The equivalence in terms of goodness of fit between the nested models was checked through four indexes: the chi-square difference test (*p* > 0.05 for Δχ^2^), normed chi-square difference (Δχ^2^/Δdf ≤ 3) and difference in the NNFI (|ΔNNFI| ≤ 0.01) and CFI (|ΔNNFI| ≤ 0.01) [48].

Regarding the second objective, the internal consistency of the factors and the total scale were calculated using Cronbach’s alpha coefficient, where α ≥ 0.70 was considered to indicate acceptable internal consistency, α ≥ 0.80 was considered to indicate good internal consistency and α ≥ 0.90 was considered to indicate excellent internal consistency [55]. These calculations were performed on the total sample of 445 family caregivers.

Regarding the third objective, the anxiety inventory scores were calculated in the total sample as index scores with values from 0 to 100, allowing three ordered categories of anxiety levels to be established: (0, 30) mild, (30, 70) medium and (70, 100) high. Moreover, a cutoff point could be stipulated, with ≥30 indicating a case and <30 indicating no case, as is customary when using indexes [57]. Beck and Steer [21] suggested 16 as cutoff for clinically significant anxiety on the BAI. A score of 16 (probable case or moderate anxiety) in the range of 0 to 63 corresponds to a score of 25.40 in the range of 0 to 100 (index score = 100 [BAI total score/63]) and a score of 26 (very probable case or severe anxiety) corresponds to 41.27. The midpoint of both values (33.33) is a score close to that stipulated (≥30) under the conventional uses of the indexes. The distribution of index scores was described through central tendency measures (arithmetic mean and median), measures of noncentral location (quartiles and deciles), measures of dispersion (sample standard deviation and semi-interquartile range) and measures of shape (coefficients of skewness and kurtosis based in moments and percentiles). The adjustment of the distribution of the index scores, as well as the item scores, to a normal distribution was tested using the Kolmogorov-Smirnov test with the Lilliefors correction.

Regarding the fourth objective, concurrent validity was verified through Pearson’s product-moment correlation coefficient (r), where r < 0.10 was considered to indicate a trivial strength of association; between 0.10 and 0.29, small; between 0.30 and 0.49, medium; between 0.50 and 0.69, high; between 0.70 and 0.89 very high; and between 0.90 and 1, unitary [58]. The significance of each correlation was tested and 95% confidence intervals were estimated using the bias-corrected and accelerated bootstrap method with the simulation of 1000 random samples due to noncompliance with the assumption of bivariate normality (Mardia’s multivariate kurtosis and skewness >2) [58]. These analyses were carried out on the total sample of 445 participants.

## 3. Results

### 3.1. Sociodemographic Characteristics of the Participants

Table 1 presents information on the family caregivers. For the total sample, the average age of the family caregivers was 32 years and they had 1–3 children. Most were women (82.5%), had a primary/secondary education (63%) and self-identified as Catholics (80.9%).

All of the patients were being treated in the hematology-oncology department and acute lymphoblastic leukemia was the most frequently occurring type of cancer. For the total sample, the average age of the children was 5.97 years (SD = 5.07), ranging from one to seven years. On average, they were hospitalized for 1.71 months (SD = 1.23) and 3.52 years (SD = 2) elapsed had since their diagnosis. The sex ratio was equal (exact probability of the two-tailed binomial test: *p* = 0.394).

### 3.2. Testing the Correlated-Factor Models Originally Proposed for the BAI in the Total Sample

Table 2 shows the GFIs for the two-, three- and four-factor models originally proposed for the BAI as well as the statistics of convergent validity for each factor (AVE and ω) and discriminant validity between factors (r^2^). All three models showed a poor fit to the data and clear problems in discriminant validity between factors (r_F1,F2_^2^ > AVE_F1_ and AVE_F2_). The data seemed to suggest a single-factor model but the single-factor model with 21 indicators also showed a poor fit. Consequently, an exploratory methodology was adopted. As a first step, the properties of the items were analyzed using the Rasch model.

### 3.3. Analysis of the Discriminability, Reliability and Normality of the Items in the Total Sample

A frequency analysis revealed that approximately 80% of the questionnaire responders selected the first two answers (1 = little or none; 2 = more or less) for all of the items, so their distributions showed positive asymmetry. The null hypothesis of univariate normality was rejected in all cases according to the Kolmogorov-Smirnov test with the Lilliefors correction.

Next, the multicollinearity of the data set was examined through the tolerance limits of the 21 items when predicting a random variable. The results indicated that item 12 (“hands trembling”) and item 13 (“shaky/unsteady”) presented excessive multicollinearity (tolerance <0.25), so they were removed from the subsequent analyses.

Table 3 presents the results of the Rasch analysis. Items 20 (“face flushed”), 16 (“fear of dying”), 1 (“numbness or tingling”), 18 (“indigestion”) and 4 (“unable to relax”) had item discrimination indexes below or close to 0.80 (<0.90 indicates weak discriminability). Except for item 4 (infit = 0.94 and outfit = 1.03), their infit and outfit indexes were also close to or above the poor fit value (1.3). These five items also had the lowest point-biserial correlation values. Due to their poor discriminability and reliability, they were removed from subsequent analyses.

Once the two items with tolerance values <0.25 and the five items that the Rasch analysis indicated had discriminability and reliability problems within the scale were eliminated, atypical multivariate cases were identified using the Mahalanobis distance measure. A total of 31 of the 445 cases had Mahalanobis *d*^2^ distances greater than 36.1 (corresponding to the 0.999 quantile in a chi-square distribution with 14 degrees of freedom) and were withdrawn. The normality of the items was re-evaluated without these atypical cases but the Kolmogorov-Smirnov test with the Lilliefors correction again indicated that the item scores did not follow a normal distribution. Because removal of the multivariate atypical cases did not reverse the univariate normality, these cases were not discarded. Following the recommendations of Tabachnick and Fidell [59] for data with strong positive asymmetry, an inverse transformation was applied to items in the total sample of 445 participants: Y = 1/(1 + X). The result revealed a monotone-increasing transformation; therefore, a higher score in the item continued to indicate a higher level of anxiety. This procedure enabled the distributions to become more symmetrical bell curves, so subsequent procedures were carried out with these transformed scores.

### 3.4. Random Division of the Total Sample into Two Subsamples

The total sample of 445 participants was randomly divided into two independent subsamples: 224 participants to explore the factorial structure and 221 participants to test the models derived from exploratory analysis. In this way, the recommendation for the sequence of exploration and confirmation of the factor model was followed. No significant differences in the sociodemographic characteristics of the family caregivers were found between the two subsamples (Table 1). There were also no statistically significant differences in the two sociodemographic variables *t*(443) = −0.457, *p* = 0.648 for age and χ²(1) = 1.20, *p* = 0.272 for sex) or two clinical variables *t*(443) = 0.496, *p* = 0.620, for time of hospitalization and *t*(443) = −0.675, *p* = 0.500, for time since diagnosis) of children. Therefore, the random division of the total sample into two subsamples did not generate biases in sociodemographic or clinical characteristics.

### 3.5. EFA of Subsample 1

To define the maximum number of factors, a parallel analysis was conducted. Two observed eigenvalues were greater than the random eigenvalues, so two factors had to be extracted according to this analysis. Extracting the factors by the principal axis and rotating the factor loadings matrix using the promax method revealed that items 14 (“fear of losing control”) and 19 (“faint/lightheaded”) had factor loadings <0.40, so they were removed from subsequent analyses. Horn’s parallel analysis was repeated, resulting in two empirical eigenvalues greater than the random eigenvalues. Therefore, the number of factors to be extracted with 12 items was also two.

When two factors were extracted again, 49.9% of the total variance was explained. After the oblique rotation, all items had factor loadings greater than 0.50 in the configuration matrix and greater than 0.60 in the structural matrix. The first factor was composed of seven physiological symptoms that involve the cardiovascular system (e.g., difficulty breathing, feeling of choking, heart pounding/racing) and the circulatory system (e.g., dizzy or lightheaded, wobbliness in legs). The second factor consisted of five symptoms of emotional problems, such as negative expectations regarding events (e.g., fear of the worst happening, unsteady), as well as intense apprehension (e.g., scared, nervous, terrified or afraid). The first factor was called “physiological symptoms,” and the second was called “emotional symptoms” (Table 4).

Both factors showed good internal consistency (α > 0.80), as did the total scale (α = 0.893). The shared variance between the two factors was 41.7%, which was lower than the AVE of the first factor (47.9%) and the second factor (49.5%); as such, the factors showed discriminant validity. Considering that the AVE values for both factors were close to 0.50 and that the composite reliability values were good (ω > 0.80), both factors were considered to have convergent validity.

### 3.6. CFA of Subsample 2

As a first step, fulfillment of the basic assumptions of multicollinearity was tested for the 221 participants in subsample 2. The results indicated no multicollinearity problems because all items had tolerance values ≥0.55. Two atypical cases with multivariate discrepancies were identified by measuring the Mahalanobis distance (d^2^ > 0.999, χ²(12) = 32.91) and were therefore withdrawn. Once these two cases were eliminated, the standardized value of Mardia’s multivariate kurtosis was 4.564, which indicates a slight deviation from multivariate normality, being less than 10 and close to 2.

Based on the EFA results, a correlated two-factor model was specified. Estimating the model parameters revealed that the residual for item 2 (“feeling hot”) had high correlations with the residuals of two items of the same factor (item 21 [“hot/cold sweats”] and item 3 [“dizzy or lightheaded”]) but item 2 had the lowest factor loading (β = 0.46), whereas the measurement weights were greater than 0.50 for the other items of the physiological symptoms factor. It was decided that item 2 would be eliminated after verifying that its removal improved the data fit and did not alter the factor’s conceptual content or measurement characteristics, such as internal consistency, which remained good (α = 0.851). Furthermore, the measurement residuals for items 11 (“feeling of choking”) and 15 (“difficulty breathing”) were correlated. Due to the content of both items and their high factor loadings, they were retained in the model and it was decided that a parameter would be freed by specifying the covariance between their residuals.

**Figure 1:**
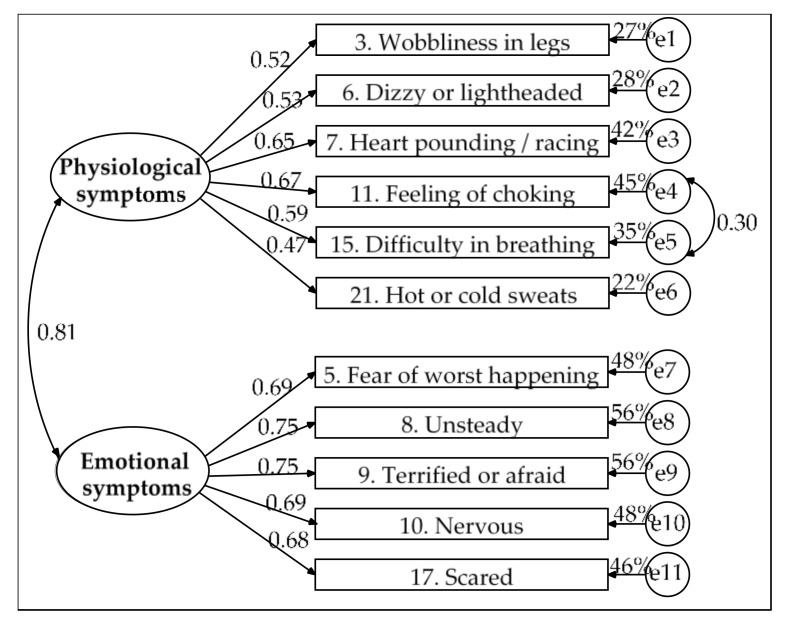
Model of two correlated factors estimated by the ML method in the subsample of 219 participants. Measurement residuals: e1 to e11.

Figure 1 presents the final model. The solution was admissible and all parameters were significant. Its goodness of fit was good (χ^2^(42) = 51.818, *p* = 0.143, χ^2^/df = 1.234, GFI = 0.959, CFI = 0.987, NFI = 0.936, RMSEA = 0.033, 90% CI (0, 0.059) and SRMR = 0.038), its parsimony was high (PR = 0.764) and its parsimony fit indexes were acceptable (PCFI = 0.754, PNFI = 0.715 and PGFI = 0.610). The emotional symptoms factor showed convergent validity (AVE = 0.508 and ω = 0.840). The physiological symptoms factor showed poor convergent validity (AVE = 0.332 and ω = 0.746). In turn, the shared variance between the two factors was very high (65%) and was higher than the AVE of each factor (33.2% for physiological symptoms and 50.8% for emotional symptoms); as such, the factors lacked discriminant validity.

The lack of discriminant and convergent validity in the physiological symptoms factor was also present when item 2 was included (*r^2^* = 0.607 > AVE = 0.328 and ω = 0.769 for physiological symptoms; *r^2^* = 0.607 > AVE = 0.508 and ω = 0.837 for emotional symptoms); thus, the model only achieved acceptable goodness of fit: χ^2^(53) = 101.396, *p* < 0.001, χ^2^/df = 1.913, GFI = 0.926, CFI = 0.941, NFI = 0.885, RMSEA = 0.065, 90% CI (0.045, 0.084) and SRMR = 0.052.

**Figure 2:**
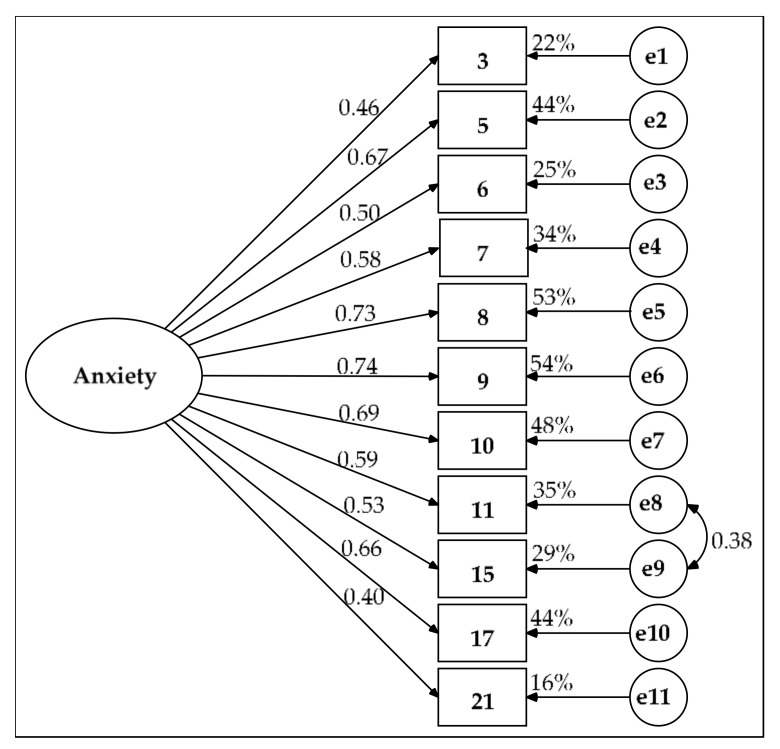
Single-factor model estimated by the maximum likelihood (ML) method in the subsample of 219 participants. Measurement residuals: e1 to e11.

Due to these problems in the model with two correlated factors, two other alternatives were pursued. The first alternative was a single-factor model with a covariance between the measurement residuals of items 11 and 15. In this model, all of the parameters were significant. Their measurement weights ranged from 0.40 to 0.74 (Figure 2). The AVE was 40.5% and the composite reliability was good (ω = 0.870). Its parsimony was high (PR = 0.782) and its parsimony fit indexes were acceptable: PNFI = 0.703, PCFI = 0.742 and PGFI = 0.607. Although goodness of fit was rejected by the chi-square test (χ^2^(43) = 81.313, *p* < 0.001), the two indexes demonstrated a good fit (χ^2^/df = 1.891 and RMSEA = 0.064, 90% CI (0.042, 0.085), *p*-close = 0.135 for H_0_: RMSEA = 0.05) and the four remaining indexes showed acceptable goodness-of-fit values (GFI = 0.932, NFI = 0.900, CFI = 0.949 and SRMR = 0.052).

**Figure 3:**
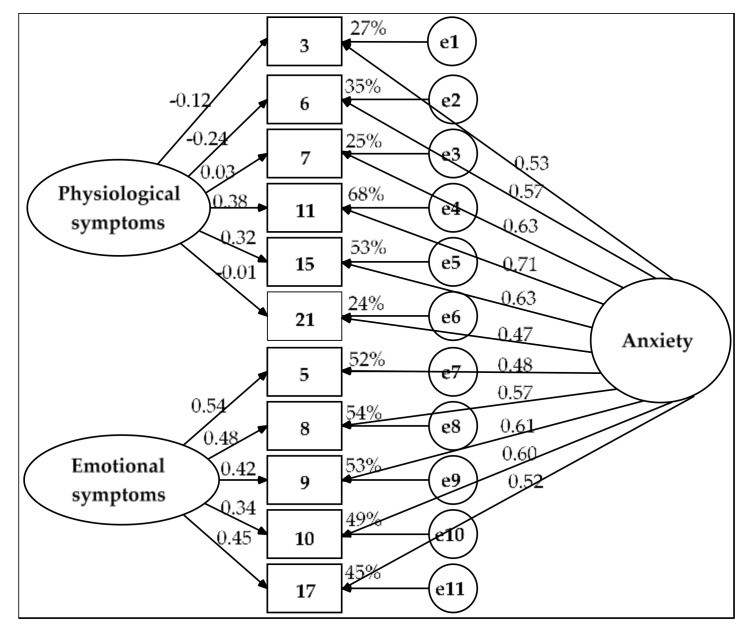
Bifactor model estimated by the ML method in the subsample of 219 participants. Measurement residuals: e1 to e11.

The second alternative was a bifactor model (Figure 3). In this model, all the residuals were independent, as were the two SFs. The solution was admissible. The goodness of fit was good (χ^2^(33) = 42.136, *p* = 0.132, χ^2^/df = 1.277, GFI = 0.966, CFI = 0.988, NFI = 0.948, RMSEA = 0.036 (90% CI = 0, 0.065) and SRMR = 0.031) and the model was equivalent to the model with the two correlated factors: Δχ^2^(Δgl= 9) = 9.682, *p* = 0.377, Δχ^2^/Δgl = 1.076 < 2, ΔGFI = 0.007, ΔCFI = 0.001 < 0.01 and ΔNFI = 0.012 ≈ 0.01. The model’s parsimony was moderate (PR = 0.600), which resulted in poor parsimonious fit indexes (PNFI = 0.569 and 0.593 < 0.60 and PGFI = 0.483 < 0.50).

The bifactor model showed convergent validity for items 5, 8, 9, 10 and 17 (AVE = 0.516 > 0.50 and ω = 0.840 > 0.70). The SF of emotional symptoms (significant measurement weights of 0.339 to 0.543, AVE_FE = 0.204 to almost 0.25 and ωh_FE = 0.328 and ECV_FE = 0.394, which are between 0.30 and 0.70) and the GF of anxiety (significant weights of 0.485 to 0.612, AVE_FG = 0.313 > 0.25 and ωh_FG = 0.512 and ECV_FG = 0.606, which are between 0.30 and 0.70) both showed substantive contributions, although the GF explained more variance in these five items than did the SF. The GF of anxiety demonstrated construct reliability (H_FG = 0.699 ≈ 0.70) but this indicator was well below 0.70 for the SF (H_FE = 0.568).

The AVE was slightly higher than 0.40 for items 3, 6, 7, 11, 15 and 21 (AVE = 0.407) and its composite reliability was merely acceptable (ω = 0.794 > 0.70); as such, its convergent validity was poor. Almost all the variance in these six items was explained by the GF of anxiety (significant measurement weights of 0.466 to 0.709, AVE_FG = 0.353 > 0.25 and ECV_FG = 0.868 and ωh_FG = 0.723 > 0.70); this factor showed construct reliability (H_FG = 0.777). The SF of physiological symptoms did not have a substantive contribution (nonsignificant measurement weights for five of the six items, AVE_FE = 0.054 < 0.25, ECV_FE = 0.132 and ωh_FE = 0.071 < 0.30); it also lacked construct reliability (H_FE = 0.267).

For the set of 11 items, the bifactor model demonstrated convergent validity by the composite reliability coefficient (ω = 0.895 > 0.70) and the AVE was greater than 0.45 (AVE = 0.457). The GF showed construct reliability (H_FG = 0.853 > 0.70) and the percentage of uncontaminated variance was less than 0.60 (PUC = 0.545). However, the contribution of the GF of anxiety was excessive (AVE_FG = 0.335 > 0.25, ωh_FG = 0.700 and ECV_FG = 0.733 ≥ 0.70). Therefore, the two SFs in the end did not have substantial weightings (AVE_FEs = 0.122 < 0.25, ωh_FEs = 0.195 and ECV_FEs = 0.267 < 0.30) and lacked construct reliability (H_FEs = 0.626 < 0.70). Consequently, CFA showed that the single-factor model was the most suitable for modeling the interrelations of the 11 selected items.

Finally, the measurement invariance of the single-factor model with 11 indicators and independent residuals was tested across the two subsamples. This multigroup analysis was performed to verify the replicability of the model without including any corrections. There were no significant differences in measurement weights between the two subsamples within the unconstrained model based on the Z-test (*p* > 0.05). There was also no difference in factor variance within the unconstrained model and the models with constraints on measurement weights and intercepts. Each of the five nested models showed acceptable goodness-of-fit values (χ^2^/df < 3, NNFI and CFI >0.90 and RMSEA and SRMR <0.08), except for the chi-square test (*p* < 0.01). The unconstrained model showed better goodness of fit than the other nested models based on the chi-square difference test (*p* < 0.01); the model with constraints on measurement weights based on Δχ^2^/df > 3; and the models with constraints on measurement intercepts, structural covariances and measurement residuals based on |ΔCFI| > 0.01. Nevertheless, the fit indexes were at least acceptable in all other comparisons: *p* > 0.01, Δχ^2^/df < 3 and |ΔNNFI| and |ΔCFI| ≤ 0.01 (Table 5). Therefore, the measurement invariance was not strict but the model showed acceptable invariance properties across two subsamples.

### 3.7. Description of Distribution of BAI-11 Index Scores and Levels of Anxiety in the Sample

Scores for the GF of anxiety (BAI-11) can be obtained through an index that varies from 0 to 100. First, the transformed scores of the items (1/(1 + X), range from 0.25 to 1) are summed. Second, the minimum possible value is subtracted from the sum. Third, this difference is divided by the difference between the maximum and minimum possible values of the sum. Finally, this quotient is multiplied by 100: Index = 100 [(sum of items—minimum possible value of the sum)/(maximum possible value - minimum possible value of the sum)]. The minimum possible value of the sum is the product of the number of items added and the minimum value of the item and its maximum possible value is the product of the number of items added and the maximum value of the item.

**Figure 4:**
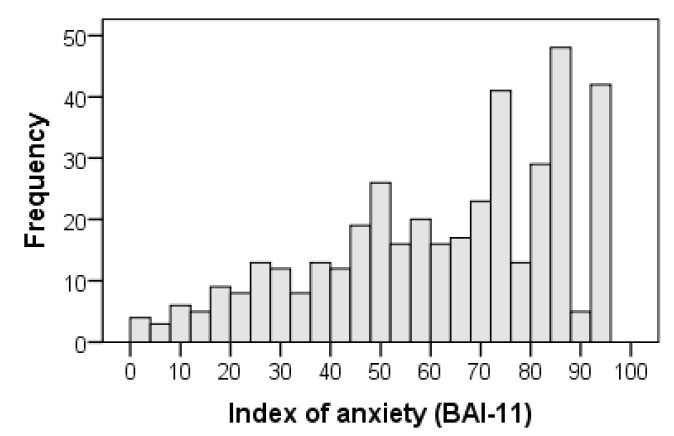
Histogram of BAI-11 index scores in the total sample of 445 participants.

The overall index of anxiety symptoms with 11 items (BAI-11) was obtained with the following formula: BAI-11 = 100 [(I3 + I5 + I6 + I7 + I8 + I9 + I10 + I11 + I15 + I17 + I21 − 2.75)/8.25]. The distribution of the BAI-11 index scores showed negative skewness (standardized value of the moment coefficient of skewness: Z_Sk_ = −4.871 < −1.96; interquartile coefficient of skewness: IQCS = −0.100) and slight deviation from mesokurtosis (standardized value of kurtosis excess: Z_K_ = −2.736 < −1.96; Kelley’s percentile coefficient of kurtosis centered at 0: PCK = 0.036, 95% CI: 0.010, 0.061). Thus, the scores did not follow a normal distribution using the Kolmogorov-Smirnov test with the Lilliefors correlation (D = 0.117, Z_D_ = 3.734, *p* < 0.001). The profile did not form a bell-shaped curve but rather corresponded to a skewed distribution with a long tail to the left. The tail of the distribution was shortened to the right but lengthened to the left, hence the contradiction between the two kurtosis coefficients. The coefficient based on moments was negative and showed platykurtosis or a shortened tail, whereas the coefficient based on percentiles was positive and showed leptokurtosis or an elongated tail (Table 6 and Figure 4).

Following common practice when index scores are used [57], a score between 0 and 29.9 was considered to indicate a low anxiety level; between 30 and 69.9, medium; and from 70 to 100, high. Based on these thresholds, 220 out of 445 family caregivers (49.4%) had high levels of total symptoms of anxiety. The central tendency (mean = 65.598, 95% bias-corrected and accelerated bootstrap (BCa) confidence interval (CI): 63.101, 68.126; median = 69.697, 95% BCa CI: 67.677, 74.748) corresponded to a medium level of anxiety (Table 6). With the stipulated cutoff point of 30, 87.6% cases of anxiety were present in the sample.

### 3.8. Concurrent Validity

The overall reliability of the BDI-II was good, similar to that of the somatic-affective symptom factor and that of its cognitive symptom factor was acceptable. The strength of the association between the BAI-11 and the BDI-II total score was high and that between the BAI-11 and the two factors of the BDI-21 was medium. These three correlations were significant and positive (Table 7).

The overall reliability of the RESI-M was excellent and that of its five factors ranged from excellent to acceptable. The BAI-11 showed significant and positive correlations with the RESI-M total score and its five factors. The strength of the association of the BAI-11 with these variables was small, except for the trivial association with social support (Table 7).

## 4. Discussion

The purpose of this research was to analyze the psychometric properties of the BAI in a population of Mexican family caregivers of children with cancer. First, we identified the factor model underlying the interrelationships of the items. The internal consistency reliability of the scale was subsequently evaluated. Then, an index was obtained to assess the level of anxiety and finally, the construct validity in relation to depression and resilience was verified.

We started by testing the correlated-factor models originally proposed for the BAI [21]. These models showed a poor fit to the data and a severe problem in discriminability between factors with two [18], three [20] or four factors [21]. Very high correlations between factors in the three models suggested a single-factor model. However, the one-factor model with 21 indicators also showed a poor fit. Therefore, we decided to explore new models.

The first exploratory step was to study the properties of the items in the total sample using the Rasch model. We chose this model because it favors unidimensionality. After eliminating two items due to excessive multicollinearity and five items due to poor discriminability and reliability properties, the sample was randomly divided. In one subsample, the factorial structure was explored and in the other subsample, the models derived from the exploratory analysis were tested.

The number of factors was determined using Horn’s parallel analysis. Two factors were found in the EFA subsample. After eliminating two more items due to low factor loadings, Horn’s analysis again indicated two factors for the remaining 12 items. The first factor, composed of physiological symptoms, consisted of seven items related to cardiovascular and circulatory changes. The second factor, composed of emotional symptoms, consisted of five items related to anxiety, nervousness and negative perceptions of surroundings. This solution coincides with that reported by Sanz et al. [25,30] and Magan et al. [27] in Spanish samples, with the exception that our solution has 12 items and the other two have 21 items. These Spanish authors identified two factors: a somatic factor, similar to this study’s physiological symptoms and an affective-cognitive factor, similar to this study’s emotional symptoms. Our solution is also very similar to the two-factor model reported by Beck et al. [18]. Nevertheless, item 8 (“unsteady”) of the somatic symptoms was interpreted as “emotional insecurity” both in this sample of Mexican family caregivers and the samples of Spanish university students [25], the general population [27] and patients with psychological disorders [30], that is, an indicator of cognitive symptoms.

Regarding the four-factor model reported by Beck et al. [20] for outpatients with anxiety disorders, the factors of neurophysiological (items 3 and 6), autonomic (items 4 and 21) and panic symptoms (items 7, 11 and 15) contain the items that make up the physiological symptom factor in the present study. The subjective anxiety factor (items 5, 9, 10 and 17) contains four out of five items that make up the emotional symptom factor in this study. However, the solutions differ in terms of the number of factors. The solution found here has two factors. This structure is more parsimonious than a four-factor model [21]. This greater parsimony is a desirable property in structural models [40,48] and it was achieved by preserving the meaning of the underlying factors. The reduction from 21 to 12 items mostly affected items pertaining to somatic symptoms, which represent 66.7% of the BAI with 21 items and 58.3% of the BAI with the 12 selected items. The removed somatic items work well in patients with panic disorders [23] but they have low discriminability and reliability in family caregivers of children with cancer.

The structure of this study also replicates the configuration found in Mexico by Galindo et al. [31] in terms of general content but with fewer elements and factors. The three factors of neurophysiological, autonomic and vasomotor symptoms in Mexican cancer patients were reduced to a physiological symptom factor in this study and the factor labeled “subjective” symptoms by Galindo et al. [31] is similar to the factor consisting of emotional symptoms in this study.

The number of BAI factors varies from one study to another [18,20,21,38,39]. However, one finds a component related to physiological changes, evaluated by two or three factors [26,29,31] and another component related to tendencies and thoughts of anxiety associated with emotional distress, the latter of which seems to be much more consistent throughout all previous studies, including Beck’s original and subsequent studies [21]. This factor is what this study calls “emotional symptoms” due to the emotional content of the items, which are related to the cognitive processes and changes associated with negative perceptions of events by individuals.

Testing the model with two correlated factors through CFA led to the elimination of item 2 (flushing). This item was eliminated because item 2 had a measurement residual with high covariances with two items of the same factor and the physiological symptoms factor had a medium effect size on this item compared to the large effect sizes it had on the other items. The goodness of fit significantly improved for the remaining 11 items and after releasing the covariance between the measurement residuals of items 11 and 15, a good fit was achieved, even according to the chi-square test. The parsimony was high, the parsimony fit indexes were acceptable and both factors demonstrated composite reliability. However, a significant problem in lack of discriminant validity among the factors was found. The high correlation between two factors suggests that they are actually one factor. In addition, the physiological symptoms factor demonstrated poor convergent validity. Both problems were found in the model that included item 2 but with acceptable values for the GFIs as opposed to good values.

Given this lack of discrimination, there are two options: specify a single-factor model or specify a bifactor model [49]. Testing of the single-factor model with the correlation between the measurement residuals of items 11 and 15 revealed that its goodness of fit was acceptable, its parsimony was high and its parsimony fit indexes were acceptable and it demonstrated construct reliability and acceptable convergent validity. The AVE was less than 0.50 but greater than 0.40, which was compensated for by a nearly excellent composite reliability [43].

It should be noted that the correlation between the residuals can be attributed to their similarity (feeling of choking and difficulty breathing), which causes them to share a specific variance outside the model that measures the physiological symptoms of anxiety (two-factor model) or the model that measures general anxiety symptoms (single-factor model).

The inclusion of corrections in the model by releasing covariance parameters between the residuals is often questioned as idiosyncratic to the sample and not clearly replicable [40]. Additional data in favor of the replicability of the single-factor model with 11 indicators (with all its independent residuals) were its adequate measurement invariance properties in the two subsamples. Consequently, this model can be validated without the need to include this correction.

Considering the existence of a GF and the possibility of improving the fit, the second alternative was to specify a bifactor model [49]. This model showed a good fit but showed worse parsimony than the previous models, resulting in poor parsimony fit indexes. Its greatest problem was an excessive contribution by the GF of anxiety to the detriment of the two SFs, mainly affecting the physiological symptoms factor, which did not have a significant contribution. While the physiological symptoms factor lacked convergent validity in the two correlated factors model, this problem arose again in the bifactor model in relation to the six items that define this factor. Therefore, the bifactor model is not a good representation of the underlying structure and highlights the true substantiality of the GF [49].

It should be noted that although two eigenvalues of the correlation matrix for the 11 items retained in the model were greater than one, upon repeating Horn’s analysis, only one empirical eigenvalue was higher than the random eigenvalues. Weighing all these data, the single-factor model was ultimately considered to be the best dimensional representation of the 11 items kept in the model, even when item 2 was included. In family caregivers of children with cancer, the physiological aspects of anxiety are intimately intertwined with the emotional and cognitive aspects. These aspects are not distinguishable as they are in patients with panic disorders [21,30]. This proposal is not novel. A single-factor model for the BAI has previously been proposed in the Australian general population [60] as well as in psychiatric inpatients and high school adolescents in the US [45].

In the studies in which a correlated-factor model is tested, the correlation between the factors is very high. In some cases, attention is not paid to these data [61,62] and in other cases, a hierarchical model is specified to maintain two, three or four factors hierarchized to a GF that have a minimal direct effect on the items [30,42,63,64]. However, when only an EFA is performed using oblique rotation, this problem of discriminant validity between factors is slightly attenuated [18] and it has no possibility of arising when an orthogonal rotation is used [31,65]. In the present study, it was decided to demonstrate the lack of discriminant validity between the factors to arrive at a single-factor solution, which was facilitated by the use of Rasch analysis applied to the items [54].

Regarding the study’s second objective, the reliability results in terms of internal consistency (estimated by Cronbach’s alpha coefficient) were satisfactory since values greater than 0.80 were obtained for the scale (with either 12 or 11 items) and its two factors. These empirical findings align with those of previous studies that reported high internal consistency indexes for the scale, ranging from α = 0.83 to α = 0.92 [18,28,30], including studies conducted in Mexico [26,29] that reported values ranging from 0.83 to 0.86.

The third objective of the study was twofold. On the one hand, it was intended to describe the distribution of BAI scores and, on the other hand, to estimate the anxiety levels in the sample. For this purpose, an overall index of anxiety symptoms was created in accordance with the single-factor model, which best represents the interrelations of the items. According to expectations [31], the distribution was not a Gaussian bell-shaped distribution. The majority of caregivers reported experiencing anxiety; therefore, the distribution was skewed. The scores were concentrated toward the right pole (high anxiety level) and a few cases far apart scattered toward the left pole (low anxiety level). Consequently, population-related norms for interpreting raw scores or within-group norms should be set based on percentile scores (scaling) and not based on standardized scores (standardizing, e.g., T-scores = 10 ([raw score—mean]/standard deviation) + 50) [36,42]. It should be noted that this type of distribution is expected when measuring a trait that is overexpressed due to some contextual factor with great influence on a population. In contrast, when the trait is determined by multiple causes with more or less equivalent effect sizes, the distribution follows a normal probability model, such as that of an expressive attitude in a free society [66,67].

Because the sampling in this study was not probabilistic and the study did not have a case-control design to set a cutoff point or classificatory thresholds, we decided to interpret the scores on the BAI-11 based on index scores. These index scores showed that approximately half of family caregivers suffered a high level of anxiety, as expected [7,8,9,11,35]. The median BAI-11 score corresponded to a medium level of anxiety, with a value close to the high anxiety threshold (≥70). Taking 30 as the cutoff point, which is a customary cutoff point when using indexes [57], approximately one out of nine family caregivers may be classified as cases of anxiety. This value corresponds to a midpoint (21 on BAI-21 equals 33.3 on BAI-11 index score) between Beck et al. [42] thresholds for a medium level (16 on BAI-21 equals 25.40 on BAI-11 index score) and a high level of anxiety (26 on BAI-21 equals 41.27 on BAI-11 index score) and to the cutoff point suggested by Sanz [36] in the Spanish adult population (19 on BAI-21 equals 30.16 on BAI-11 index score).

The fourth objective was to verify construct validity in relation to depression and resilience. The hypothesis of the direct relationship between anxiety and depression with a moderate to high strength of association was supported [7,11]. Furthermore, the expectation of an inverse relationship between anxiety and resilience was met, with a weak strength of association [37,38,39]. Consequently, this study provides construct validity evidence for this short, one-dimensional version of the BAI.

One limitation of this study is the use of nonprobability sampling; therefore, caution should be used when interpreting the results. A second limitation is the cross-sectional design, making it impossible to estimate the temporal reliability or to test the temporal stability of the factor model. The ratio of the sexes was very unequal (78:367, approximately one man for every five women), which is characteristic of the population and the number of men was less than 100. These two conditions prevented us from testing the measurement invariance of the single-factor model across women and men. A fourth limitation is the hospital setting in which the data were collected; thus, the generalizability of the results to other clinical settings should be approached with care.

## 5. Conclusions

In this sample of Mexican family caregivers of children with cancer, the original correlated-factor models for the 21 items were not supported by the data. They presented a poor fit to the data and severe problems in discriminant validity that suggested a single-factor model. The EFA revealed two factors that are consistent with the original two-factor model and have a strong correlation. However, the CFA again showed a severe problem in discriminant validity that was not solved when defining a bifactor model with a GF of anxiety and two SFs of physiological and emotional symptoms. A single-factor model reduced to 11 items attained acceptable goodness of fit, high parsimony, acceptable parsimony fit indexes, convergent validity, good internal consistency reliability and construct validity in relation to depression and resilience. A unidimensional model was the best model for this sample, indicating that emotional and physiological symptoms are intimately interwoven in the anxiety experienced by family caregivers of children with cancer. The BAI-11 was scored as an index with values ranging from 1 to 100. The distributions of its scores were skewed to the left and did not follow a normal distribution; therefore, the BAI-11 index scores based on the norms relative to the population of Mexican family caregivers of children with cancer should be interpreted through percentile scores. Based on an absolute interpretation using common thresholds for indexes, a high level of anxiety (≥70) was observed in approximately half of the caregivers; in addition, approximately one to nine family caregivers were classified as possible cases of anxiety (≥30). Consequently, it is necessary to implement strategies to reduce anxiety in this population.

We suggest testing the measurement invariance of the single-factor model with 11 indicators across women and men with two samples balanced in size and with at least 200 participants, which would constitute important construct validity evidence. Further research could test this model in other populations, contexts and cultures in which families are caring for children with cancer. This study has clinical utility in having achieved a short, one-dimensional instrument for measuring anxiety with a simple interpretation procedure. However, norm-based scoring (quantile or standard ten scores) could be established using probability sampling as well as a cutoff value to define clinical cases using a gold standard (e.g., the Structured Clinical Interview for DSM-5 [68]).

## Figures and Tables

**Table 1:** Sociodemographic data of the family caregivers.

Variable	Est.	Total Sample	Subsample 1	Subsample 2	Hypothesis Test
		(*n* = 445)	(*n* = 224)	(*n* = 221)	
Age	M	32.26	32.4	32.1	*t* = 0.341
	±SD	±8.65	±8.8	±8.85	df = 443
					*p* = 0.733
Number of children	M	2.32	2.37	2.27	*t* = 0.847
	±SD	±1.23	±1.26	±1.21	df = 443
					*p* = 0.397
Sex					χ² = 0.868
Male	*n* (%)	367 (82.5)	181 (80.1)	186 (84.2)	df = 1
Female	*n* (%)	78 (17.5)	43 (19.2)	35 (15.8)	*p* = 0.351
Parental role					
Father	*n* (%)	367 (82.5)	181 (80.1)	186 (84.2)	df = 1
Mother	*n* (%)	78 (17.5)	43 (19.2)	35 (15.8)	*p* = 0.351
Education					U = 24.538
No education	*n* (%)	15 (3.4)	7 (3.1)	8 (3.6)	Z_U_ = −0.167
Prim. & secondary	*n* (%)	280 (62.9)	139 (62.1)	141 (63.8)	*p* = 0.867
Preparatory	*n* (%)	115 (25.8)	59 (26.3)	56 (25.3)	
University	*n* (%)	35 (7.9)	19 (8.2)	16 (7.2)	
Religion					χ² = 1.42
Catholic	*n* (%)	360 (81.1)	180 (80.7)	180 (81.4)	df = 2
Christian	*n* (%)	52 (11.7)	24 (10.8)	28 (12.7)	*p* = 0.491
No religion	*n* (%)	32 (7.2)	19 (8.5)	13 (5.9)	

Note. Descriptive statistics: M = arithmetic mean, SD = standard deviation, *n* = simple absolute frequency and % = percentage. Comparison tests: *t* = Student’s *t*-test for two independent samples, U = Mann-Whitney U-test, χ² = chi-square test for homogeneity between two independent samples, df = degrees of freedom and *p* = probability value of a two-tailed test. Z_U_ = standardized value of the Mann-Whitney U statistic that follows a standard normal distribution under the null hypothesis (H_0_: mean range in Y of group 1 = mean range in Y of group 2) when the total sample size (n = n_1_ + n_2_) tends to infinity (asymptotic approximation).

**Table 2:** Fit indexes of the one-factor model and the correlated-factor models originally proposed for the BAI as well as the AVE, shared variance and composite reliability of their factors.

Index	Factor	1F	Correlated-Factor Models			Shared Variance (r^2^)		
			2F	3F	4F	F2	F3	F4
χ^2^		1031.017	894.327	908.672	824.715			
df		189	188	186	183			
*p*-value		<0.001	<0.001	<0.001	<0.001			
χ^2^/df		5.455	4.757	4.885	4.507			
GFI		0.794	0.836	0.831	0.846			
AGFI		0.748	0.798	0.791	0.806			
NFI		0.797	0.824	0.821	0.838			
CFI		0.827	0.855	0.852	0.869			
RMSEA (90% CI)		0.099 (0.093, 0.105)	0.091 (0.085, 0.097)	0.093 (0.087, 0.099)	0.088 (0.082, 0.094)			
SRMR		0.063	0.059	0.060	0.057			
Average variance extracted (AVE)	GF = Anxiety	0.422						
	F1 = Somatic		0.429			0.759		
	F2 = Cognit.		0.489					
	F1 = Somatic			0.415		0.767	0.922	
	F2 = Subject.			0.522			0.731	
	F3 = Panic			0.449				
	F1 = Neuroph.				0.451	0.854	0.924	0.750
	F2 = Subject.				0.522		0.733	0.453
	F3 = Panic				0.450			0.752
	F4 = Autono.				0.431			
McDonald’s omega or composite reliability	GF = Anxiety	0.938						
	F1 = Somatic		0.912					
	F2 = Cognit.		0.869					
	F1 = Somatic			0.885				
	F2 = Subject.			0.867				
	F3 = Panic			0.762				
	F1 = Neuroph.				0.850			
	F2 = Subject.				0.867			
	F3 = Panic				0.762			
	F4 = Autono				0.750			

Note. 1F = one-factor model: GF = general factor of anxiety (items 1–21). Correlated-factor models: two-factor: F1 = somatic symptoms with 14 indicators (items 1, 2, 3, 6, 7, 8, 11, 12, 13, 15, 18, 19, 20 and 21) and F2 = cognitive symptoms with seven indicators (items 4, 5, 9, 10, 14. 16 and 17). three-factors: F1 = somatic symptoms with 11 indicators (items 1, 2, 3, 6, 8, 12, 13, 18, 19, 20 and 21), F2 = subjective symptoms with six indicators (items 4, 5, 9, 10, 14 and 17) and F3 = panic symptoms with four indicators (items 7, 11, 15 and 16). four-factors: F1 = neurophysiological symptoms with seven indicators (items 1, 3, 6, 8, 12, 13 and 19), F2 = subjective symptoms with six indicators (items 4, 5, 9, 10, 14 and 17), F3 = panic symptoms with four indicators (items 7, 11, 15 and 16) and F4 = autonomic symptoms with four indicators (items 2, 18, 20 and 21). Method: maximum likelihood method. Sample size (*n*) = 455.

**Table 3:** Rasch analysis of 19 Beck Anxiety Inventory (BAI) items in the total sample (*n* = 445).

Items	Infit	Outfit	r_pb_	Discrim.
11. Feeling of choking	1.09	0.79	0.65	1.12
15. Difficulty breathing	1.27	0.91	0.59	1.05
20. Face flushed	1.27	1.30	0.49	0.81
21. Hot/cold sweats	1.18	0.94	0.62	1.04
2. Feeling hot	1.13	1.18	0.52	0.85
9. Terrified or afraid	1.02	0.80	0.68	1.15
14. Fear of losing control	0.96	0.89	0.68	1.13
6. Dizzy or lightheaded	1.03	1.10	0.62	0.97
16. Fear of dying	1.50	1.50	0.51	0.75
1. Numbness or tingling	1.15	1.31	0.51	0.73
7. Heart pounding/racing	0.96	0.80	0.70	1.16
18. Indigestion	1.22	1.32	0.54	0.77
3. Wobbliness in legs	0.92	0.94	0.62	0.98
17. Scared	1.02	0.94	0.64	0.99
8. Unsteady	0.83	0.84	0.68	1.08
19. Faint/lightheaded	0.88	0.79	0.68	1.10
4. Unable to relax	0.94	1.03	0.58	0.76
10. Nervous	0.68	0.75	0.71	1.10
5. Fear of worst happening	1.02	1.08	0.58	0.85

Note. Infit = inlier-sensitive fit index, Outfit = outlier-sensitive fit index, r_pb_ = point-biserial correlation coefficient and Discrim. = index of empirical discrimination.

**Table 4:** Factorial structure of 12 BAI items explored in the subsample of 224 participants.

Items	h^2^	Pattern Matrix		Structure Matrix	
		F1	F2	F1	F2
2. Feeling hot	0.500	0.844	−0.254	0.680	0.292
15. Difficulty breathing	0.523	0.731	−0.013	0.723	0.460
21. Hot/cold sweats	0.515	0.728	−0.016	0.718	0.455
7. Heart pounding/racing	0.554	0.611	0.185	0.731	0.580
11. Feeling of choking	0.479	0.597	0.135	0.684	0.521
6. Dizzy or lightheaded	0.487	0.572	0.174	0.685	0.544
3. Wobbliness in legs	0.396	0.527	0.144	0.620	0.484
17. Scared	0.582	−0.180	0.867	0.380	0.751
5. Fear of the worst happening	0.449	−0.054	0.704	0.401	0.669
10. Nervous	0.478	0.042	0.664	0.471	0.691
9. Terrified or afraid	0.519	0.173	0.596	0.559	0.708
8. Unsteady	0.501	0.178	0.580	0.553	0.695
Cronbach’s alpha		0.864	0.829		
Average variance extracted				0.479	0.495
McDonald’s omega				0.865	0.830
Cronbach’s alpha for the total scale		0.893			
Correlation between factors		0.646			
Total explained variance		49.85%			

Note. Extraction method: principal axis. Rotation method: promax. h^2^ = commonality of extraction. F1 = factor of physiological symptoms and F2 = factor of emotional symptoms of anxiety.

**Table 5:** Measurement invariance across two subsamples for the single-factor model with 11 indicators and independent residuals.

Nested Models	Fit Indexes	Thresholds		Nested Models				
		Close	Accept.	U	MW	MI	SC	MR
	χ^2^			229.894	260.456	279.254	279.782	302.051
	df			88	98	109	110	121
	*p*	>0.05	>0.01	<0.001	<0.001	<0.001	<0.001	<0.001
	χ^2^/df	≤2	≤3	2.612	2.658	2.562	2.543	2.496
	NNFI	≥0.95	≥0.90	0.918	0.915	0.920	0.921	0.924
	CFI	≥0.95	≥0.90	0.934	0.925	0.921	0.921	0.916
	RMSEA	≤0.05	<0.08	0.060	0.061	0.059	0.059	0.058
	90% CI (LI, LS)			(0.050, 0.069)	(0.051, 0.070)	(0.049, 0.068)	(0.049, 0.067)	(0.048, 0.067)
		
	SRMR	≤0.05	<0.10	0.056	0.063	0.062	0.066	0.077
U	Δχ^2^				30.562	49.36	49.888	72.157
	Δdf				10	21	22	33
	*p*	>0.05	>0.01		0.001	<0.001	0.001	<0.001
	Δχ^2^/df	≤2	≤3		3.056	2.350	2.268	2.187
	ΔNNFI	≤0.01			0.002	0.003	0.004	0.006
	ΔCFI	≤0.01			0.010	0.013	0.013	0.018
MW	Δχ^2^					18.798	19.326	41.595
	Δdf					11	12	23
	*p*	>0.05	>0.01			0.065	0.081	0.010
	Δχ^2^/df	≤2	≤3			1.709	1.611	1.808
	ΔNNFI	≤0.01				0.005	0.006	0.008
	ΔCFI	≤0.01				0.004	0.003	0.009
MI	Δχ^2^						0.528	22.797
	Δdf						1	12
	*p*	>0.05	>0.01				0.467	0.029
	Δχ^2^/df	≤2	≤3				0.528	1.900
	ΔNNFI	≤0.01					0.001	0.003
	ΔCFI	≤0.01					<0.001	0.005
SC	Δχ^2^							22.269
	Δdf							11
	*p*	>0.05	>0.01					0.022
	Δχ^2^/df	≤2	≤3					2.024
	ΔNNFI	≤0.01						0.002
	ΔCFI	≤0.01						0.005

Note. Models nested in constraints. U = unconstrained, MW = measurement weight, MI = measurement intercept, SC = structural covariance and MR = measurement residual. Thresholds for the interpretation of goodness of fit are shown in the second (close fit) and third (acceptable fit) columns.

**Table 6:** Descriptive statistics of BAI-11 index scores and levels of anxiety.

Descriptive Statistics	BAI-11 Index Score	Percentile	BAI-11 Index Score	Level of Anxiety	*n* (%)
Min	0	P10	26.263	Low (0–29.9)	55 (12.4%)
Max	100	P20	42.424	Medium (30–69.9)	170 (38.2%)
M	65.598	P25	47.475	High (70–100)	220 (49.4%)
SD	25.643	P30	51.515		
SIQR	20.202	P40	61.616		
Sk	−0.565	P50	69.697		
SEK	0.116	P60	75.758		
IQCS	−0.100	P70	83.838		
K	−0.632	P75	87.879		
SEK	0.231	P80	87.879		
PCK	0.036	P90	93.939		

Note. Sample size: *n* = 445. BAI-11 index score = index of symptoms of anxiety = 100 [(I3 + I5 + I6 + I7 + I8 + I9 + I10 + I11 + I15 + I17 + I21 − 2.75)/8.25]. Descriptive statistics: Min = sample minimum value, Max = sample maximum value, M = sample arithmetic mean, SD = sample standard deviation, SIQR = semi-interquartile range, Sk = Fisher-Pearson moment coefficient of skewness, SEK = Fisher’s standard error of coefficient of skewness, IQCS = Bowley’s interquartile coefficient of skewness, K = Fisher’s kurtosis excess, SEK = Fisher’s standard error of coefficient of kurtosis excess and PCK = Kelley’s percentile coefficient of kurtosis centered at 0 (standard error = 0.278/√445 = 0.013). Levels of anxiety: *n* = simple absolute frequency and % = simple percentage.

**Table 7:** Correlations between the BAI-11 and the two validity criteria (BDI-II and RESI-M).

Concurrent Validity Criteria	NI	α	BAI-11
			r (95% CI)
BDI-II	21	0.893	−0.526 *** (−0.601, −0.436)
Somatic-affective symptoms	12	0.856	−0.473 *** (−0.552, −0.389)
Cognitive symptoms	9	0.794	−0.498 *** (−0.579, −0.403)
RESI-M	43	0.948	0.272 *** (0.176, 0.374)
Strength and self-confidence	19	0.932	0.274 *** (0.178, 0.377)
Social competence	8	0.859	0.215 *** (0.118, 0.304)
Family support	6	0.881	0.229 *** (0.134, 0.334)
Social support	5	0.903	0.090 * (0.002, 0.190)
Structure	5	0.752	0.137 ** (0.052, 0.229)

Note. BDI-II = Beck Depression Inventory, second edition. RESI-M = Measurement Scale of Resilience. Statistics: NI = number of items, α = Cronbach’s alpha, r = Pearson’s product-moment correlation coefficient, 95% CI = 95% confidence interval estimated by the bias-corrected and accelerated (BCA) bootstrap method with the simulation of 1000 random samples and probability values of two-tailed tests through BCA: not significant = ^ns^ *p* > 0.05, * *p* ≤ 0.05, ** *p* ≤ 0.01, *** *p* ≤ 0.001.
